# Efficacy and Safety of Fruquintinib Plus PD-1 Inhibitors Versus Regorafenib Plus PD-1 Inhibitors in Refractory Microsatellite Stable Metastatic Colorectal Cancer

**DOI:** 10.3389/fonc.2021.754881

**Published:** 2021-10-06

**Authors:** Liying Sun, Shenglan Huang, Dan Li, Ye Mao, Yurou Wang, Jianbing Wu

**Affiliations:** ^1^ Department of Digestive Oncology, The Second Affiliated Hospital of Nanchang University, Nanchang, China; ^2^ Jiangxi Key Laboratory of Clinical and Translational Cancer Research, Nanchang, China; ^3^ Department of Oncology, The First Affiliated Hospital of Nanchang University, Nanchang, China

**Keywords:** colorectal cancer, immunotherapy, microsatellite stable, fruquintinib, regorafenib, PD-1

## Abstract

**Background:**

Microsatellite stability (MSS) or mismatch repair proficient (pMMR) metastatic colorectal cancer (mCRC) is resistant to immune checkpoint inhibitors. Studies have shown that antiangiogenic drugs combined with programmed death receptor-1 (PD-1) inhibitors can improve immunosuppression. The purpose of this study was to compare the efficacy of fruquintinib combined with PD-1 inhibitor (FP) and regorafenib combined with PD-1 inhibitor (RP) in the treatment of advanced mCRC with MSS or pMMR.

**Materials and Methods:**

We retrospectively collected advanced MSS or pMMR mCRC patient data from The Second Affiliated Hospital of Nanchang, China, from June 2019 to March 2021. Then, we analyzed and compared the efficacy and safety of FP and RP.

**Results:**

A total of 51 patients who met the criteria were divided into FP (n = 28) and RP groups (n = 23). The overall response rate of the FP and RP groups was 7.1% and 8.7% and the disease control rate was 89.3% and 56.5%, respectively. The median progression-free survival (PFS) time was higher in the FP group than in the RP group (6.4 vs. 3.9 months, respectively; P = 0.0209). Patients with no liver metastasis, KRAS wild type, and left colon tumor may benefit from FP. Eight patients (15.7%) had grade 3 toxicity related to treatment. Cox multivariate regression analysis showed that the treatment method was an independent risk factor for median PFS time.

**Conclusion:**

Our study indicates that FP could improve PFS time of patients with advanced mCRC compared with RP.

## Introduction

Global colorectal cancer (CRC) ranks third in morbidity and second in mortality (1). For patients with advanced CRC who have failed to receive standard first-line and second-line treatment, the third-line treatment consists of regorafenib, fruquintinib, and TAS-102 (2), but the effects are not suitable (3). In recent years, the application of immune checkpoint inhibitor (ICI) has brought new hope for improving the therapeutic effect of metastatic CRC (mCRC) treatment (4–6).

As monoclonal antibodies (mAb) to programmed death receptor-1 (PD-1), pembrolizumab and nivolumab have shown considerable activity in advanced CRC patients with high microsatellite instability (MSI-H) or DNA mismatch repair defects (dMMR) tumors (7–9). However, MSI-H or dMMR mCRC patients account for only 5% of all CRC patients. Ninety-five percent of CRC patients with microsatellite stability (MSS) or mismatch repair proficient (pMMR) CRC do not respond to immunotherapy (10), which is a key clinical problem related to PD-1 inhibitors. Zelenay et al. found that immunotherapy combined with antivascular endothelial growth factor therapy can improve the immunosuppressive state in a CRC mouse model (11, 12). Clinical studies have shown that antiangiogenic drugs combined with immune checkpoint blocking can significantly improve the effectiveness of malignant tumor treatment (13–16). Therefore, ICI combined with antiangiogenesis therapy may overcome the resistance of MSS or pMMR mCRC to immunotherapy.

Fruquintinib is an effective and highly selective oral inhibitor of vascular endothelial growth factor receptor (VEGFR) 1, 2, 3 tyrosine kinase (17, 18). The FRESCO trial—a multicenter, randomized, double-blind, placebo-controlled phase III trial—compared fruquintinib with placebo in patients with mCRC who failed to receive standard chemotherapy (19). The results showed that the median overall survival (OS) and the median progression-free survival (PFS) of the patients receiving fruquintinib were 9.3 and 3.71 months, respectively, which were significantly longer than those of patients in the placebo control group. Clinical studies have shown that fruquintinib has the advantages of low off-target toxicity, good drug resistance, and strong curative effect. Regorafenib is a new type of multitarget tyrosine kinase inhibitor, which can inhibit the activation of VEGFR-1, VEGFR-2, VEGFR-3, FGFR, PDGFR, KIT, RET, TIE2, and BRAF (20). The results of CORRECT—an international, multicenter, phase III clinical study—showed that the median OS of the regorafenib group was 6.4 months, which was significantly longer than that of the placebo group (21). A subsequent CONCUR study conducted in Asia showed that regorafenib significantly prolonged the median OS to 8.8 months in patients with advanced CRC (22), thus indicating that regorafenib can improve the survival of patients with refractory mCRC. Fruquintinib and regorafenib are both third-line drugs for advanced CRC; indirect comparison by meta-analysis showed no significant difference in their efficacy and safety in advanced CRC (23–28).

In a phase Ib clinical study from Japan, the objective response rate (ORR) of regorafenib combined with nivolumab in patients with refractory mCRC was 36% and the median PFS was 7.9 months (29). The results of this early trial gave hope to patients and oncologists worldwide and provided additional options for patients with refractory mCRC. However, a retrospective study of 18 patients at the American Cancer Center failed to reveal comparable clinical activity of regorafenib plus nivolumab (30). In a retrospective clinical study of regorafenib combined with anti-PD-1 antibody in the treatment of MSS or pMMR mCRC patients in China, some potential benefits in disease control rate (DCR) and PFS were observed, albeit the results showed no objective effect (31). Therefore, additional evidence is needed to evaluate this joint strategy. Reportedly, a patient with advanced MSS CRC showed a rapid response after the failure of multiline therapy when fruquintinib was combined with anti-PD-1; then, the effect of fruquintinib combined with anti-PD-1 was verified in a CT26 cell (MSS) mouse co-gene model (32). Studies have shown that fruquintinib combined with PD-1 inhibitors can synergistically inhibit the progression of CRC, change the tumor microenvironment, and contribute to antitumor immune response. In a study conducted in China, the ORR and DCR of regorafenib or fruquintinib combined with camrelizumab in the treatment of MSS or pMMR mCRC patients were 25.0% and 62.5%, respectively, reflecting the good efficacy of regorafenib or fruquintinib plus camrelizumab in MSS or pMMR mCRC patients and thereby indicating the potential of ICI combined therapy (33).

There are no studies comparing the efficacy and safety of fruquintinib combined with PD-1 inhibitors against those of regorafenib and PD-1 inhibitors in the treatment of advanced CRC. Therefore, in this study, we retrospectively analyzed the treatment of refractory mCRC patients in the second affiliated Hospital of Nanchang University in China and compared the efficacy and safety of fruquintinib combined with PD-1 inhibitors against those of regorafenib combined with PD-1 inhibitors in the treatment of advanced CRC.

## Patients and Methods

### Patients

We conducted a retrospective study of patients with advanced MSS or pMMR CRC treated in the second affiliated Hospital of Nanchang University. The patients received fruquintinib or regorafenib combined with PD-1 inhibitors as third-line or above posterior line therapy for a compassionate purpose. Formalin-fixed paraffin-embedded tissue specimens were used to detect the protein expression deletion of four kinds of MMR (MLH1/MSH2/MSH6/PMS2) by immunohistochemistry (IHC), or five tumor microsatellite sites [five single nucleotide sites (BAT25, BAT26, NR21, NR24, Mono27)] were analyzed by polymerase chain reaction (PCR) to determine the MMR/MSI status of the tumor. The main selection criteria included 1) advanced or mCRC that was histologically or cytologically confirmed to be at least refractory to second-line system treatment or could not tolerate standard treatment, 2) age 18–79 years (3), performance status of (ECOG PS) 0–2 in the Eastern Cancer Cooperation group, 4) adequate bone marrow reserve, 5) adequate liver and kidney function, and 6) at least one measurable lesion based on RECIST v1.1. The main exclusion criteria included 1) history of active, chronic, or recurrent autoimmune diseases and 2) severe complications. This study was conducted in accordance with the Helsinki Declaration and approved by the Ethics Review Committee of the Second Affiliated Hospital of Nanchang University.

### Treatment Methods

Patients in the FP group took 3–5 mg oral fruquintinib, and patients in the RP group took 80–160 mg oral regorafenib once a day for 21 consecutive days in 28-day cycles. In order to control the side effects associated with treatment, some patients had adjusted dosages. Regarding immunotherapy, the patients were injected intravenously with PD-1 inhibitors at the recommended dose from the first day of taking molecular targeted drugs: toripalimab (240 mg) every 3 weeks, nivolumab (200 mg) every 2 weeks, and sintilimab or camrelizumab (200 mg) every 3 weeks.

### Efficacy and Toxicities

The tumor was measured by computed tomography every 2–3 cycles of immunotherapy, and the tumor response was evaluated according to RECIST version 1.1 until the disease progressed or subsequent treatment began. Tumor remission was defined as complete remission (CR), partial remission (PR), stable disease (SD), or progression of disease (PD). ORR is defined as the best percentage of patients with total remission in either CR or PR. DCR was defined as the proportion of patients with the best overall response to CR, PR, or SD. PFS was calculated from the beginning of treatment to the time of disease progression or death from any cause. The toxicity assessment was based on the National Cancer Institute General Toxicity Standard version 5.0 (CTC5.0). The deadline for data was June 20, 2021.

### Statistical Analysis

A Pearson chi-square test or Fisher exact test was used to compare the classification variables in the baseline features. The mean standard deviation (SE) was used to describe the variable distribution in the normal distribution, and the median and range were used to describe the variable distribution in the non-normal distribution. The Kaplan–Meier method was used for survival analysis, and the logarithmic rank test was used for the difference of survival curve. The Cox regression model was used to analyze the variables with *P <*0.05 in univariate analysis. The hazard ratio (HR) and confidence interval (CI) were calculated. *P <*0.05 was considered to be statistically significant. All data were analyzed using SPSS 26.0 software and GraphPad Prism 8.0.

## Results

### Patient Characteristics

**Figure 1:**
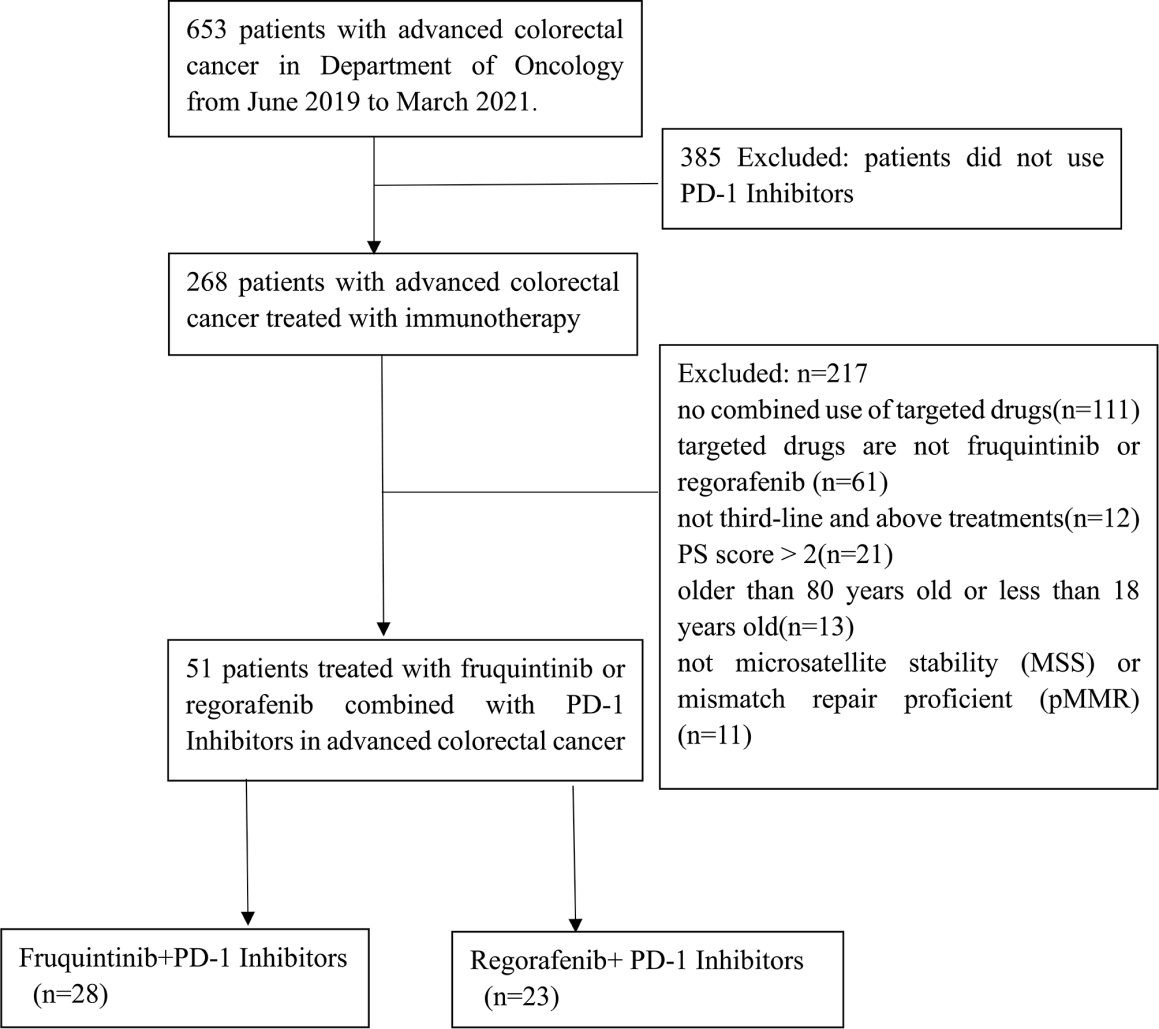
Flowchart of the patient queue.

We included 653 patients who were diagnosed with mCRC from June 2019 to March 2021 at the Second Affiliated Hospital of Nanchang University. Among them, 268 mCRC patients were selected according to the inclusion criteria. Finally, 51 mCRC patients treated with fruquintinib or regorafenib combined with PD-1 inhibitors were enrolled in the present study (**Figure 1**). The deadline for data was June 20, 2021, with a median follow-up period of 6.2 months (IQR 3.9–8.43).

**Table 1** shows the baseline characteristics of patients. Among them, 28 mCRC patients received FP, and the other 23 mCRC patients received RP. In this study, these patients were treated with FP or RP as the third line (56.9%) or more than third line (43.1%) of mCRC treatment. All patients experienced tumor progression after standard chemotherapy. Thirty-nine patients (76.5%) had left primary tumor, 12 patients (23.5%) had right primary tumor, and 38 patients (74.5%) had liver metastasis. As for primary tumor gene mutations, 14 patients had a RAS mutation, 1 patient had a BRAF mutation, and 18 patients had RAS/BRAF wild type. In the combined therapy, the initial doses of fruquintinib were 3 mg (20 patients), 4 mg (5 patients), and 5 mg (3 patients); as for regorafenib, 17 patients started with 80 mg, 5 patients started with 120 mg, and 1 patient started with 160 mg. The types of PD-1 inhibitors used were sintilimab, camrelizumab, toripalimab, and nivolumab. PD-1 inhibitors sintilimab (53.6%) and camrelizumab (46.4%) were used in the FP group, and camrelizumab (52.2%) was the most common PD-1 inhibitor in the RP group. In the FP group, the cycles of PD-1 inhibitors plus fruquintinib ranged from 3 to 8, with a median of 6. In the RP group, the cycles of PD-1 inhibitor plus regorafenib were 3 to 5, with a median of 4.

**Table 1:** Baseline clinical characteristics of patients.

Characteristic	Total, *n* (%)	FP group, *n* (%)	RP group, *n* (%)	*P-*value
Patients, *N* (%)	51	28	23	
Median age (range)	54.2 ± 11.9	54.6 ± 11.7	53.0 ± 12.02	0.724
Age group				0.718
<65 years	41 (80.4)	22 (78.6)	19 (82.6)	
≥65 years	10 (19.6)	6 (21.4)	4 (17.4)	
Sex				0.304
Male	27 (52.9)	13 (46.4)	14 (60.9)	
Female	24 (47.1)	15 (53.6)	9 (39.1)	
Baseline ECOG PS				0.702
0	21 (41.2)	13 (46.4)	8 (34.8)	
1	22 (43.1)	11 (39.3)	11 (47.8)	
2	8 (15.7)	4 (14.3)	4 (17.4)	
Time from first diagnosis to randomization, median (range), months	24 (16.0–47.0)	22 (15.3–39.5)	26 (18.0–50.0)	0.35
Time from first metastatic diagnosis to randomization				0.276
<18 months	15 (29.4)	10 (35.7)	5 (21.7)	
≥18 months	36 (70.6)	18 (64.3)	18 (78.3)	
Primary disease site at first diagnosis				0.18
Colon	28 (54.9)	13 (46.4)	15 (65.2)	
Rectum	23 (45.1)	15 (53.6)	8 (34.8)	
Colon and rectum	51 (100.0)	28 (100.0)	23 (100.0)	
Primary tumor location at first diagnosis				0.11
Left	39 (76.5)	19 (67.9)	20 (87.0)	
Right	12 (23.5)	9 (32.1)	3 (13.0)	
Left and right	51 (100.0)	28 (100.0)	23 (100.0)	
Multiple metastases				
Liver	38 (74.5)	18 (64.3)	20 (87.0)	0.065
Lung	43 (84.3)	24 (85.7)	19 (82.6)	0.762
Peritoneum	13 (25.5)	7 (25.0)	6 (26.1)	0.929
Previous treatment agents				
5-Fluorouracil	43 (84.3)	24 (85.7)	18 (78.3)	0.487
Oxaliplatin	47 (92.2)	26 (92.9)	21 (91.3)	0.837
Irinotecan	48 (94.1)	26 (92.9)	22 (95.7)	0.673
Bevacizumab	40 (78.4)	20 (71.4)	20 (87.0)	0.18
Cetuximab	20 (39.2)	14 (50.0)	6 (26.1)	0.082
Regorafenib	9 (17.6)	3 (10.7)	6 (26.1)	0.152
Fruquintinib	0	0	0	
Number of prior treatment lines on metastatic disease				0.964
3	29 (56.9)	16 (57.1)	13 (56.5)	
>3	22 (43.1)	12 (42.9)	10 (43.5)	
Prior antitumor treatment				
Chemotherapy and pharmacological treatment	51 (100.0)	28 (100.0)	23 (100.0)	1
Radiation therapy	9 (17.6)	5 (17.9)	4 (17.4)	0.965
Surgery	45 (88.2)	23 (82.1)	22 (95.7)	0.136
Gene mutation status				0.853
RAS/BRAF wild type	18 (35.3)	13 (46.4)	5 (21.7)	
RAS mutant	14 (27.5)	10 (35.7)	4 (17.4)	
BRAF mutant	1 (1.9)	0 (0.0)	1 (4.3)	
Unknown	18 (35.3)	5 (17.9)	13 (56.5)	
Prior chemotherapy with VEGF and EGFR inhibitors				0.348
Neither	2 (4.0)	1 (3.6)	1 (4.3)	
VEGF only	28 (54.9)	12 (42.9)	16 (69.6)	
EGFR only	8 (15.7)	6 (21.4)	2 (8.7)	
Both	12 (23.5)	8 (28.6)	4 (17.4)	
Unknown	1 (1.9)	1 (3.6)	0 (0.0)	
PD-1 cycles	5 (3–8)	6 (3–8)	4 (3–5)	0.105

PD-1, programmed death receptor-1; FP, fruquintinib combined with PD-1 inhibitors; RP, regorafenib combined with PD-1 inhibitors; ECOG PS, Eastern Cooperative Oncology Group performance status; VEGF, vascular endothelial growth factor; EGFR, epidermal growth factor receptor.

### Clinical Efficacy

**Figure 2:**
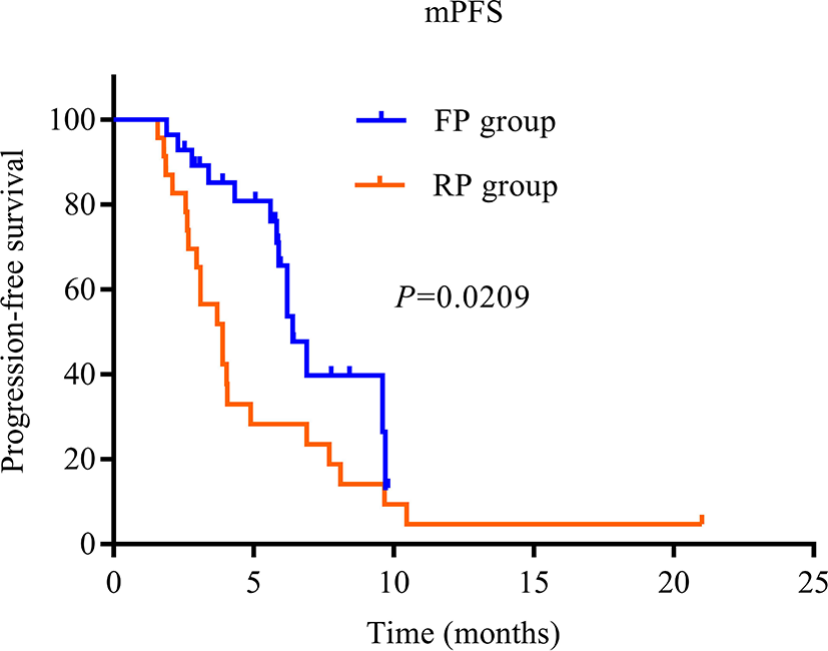
Kaplan–Meier curves of median progression-free survival of patients in the two groups. PD-1, programmed death receptor-1; FP, fruquintinib combined with PD-1 inhibitors; RP, regorafenib combined with PD-1 inhibitors; PFS, progression-free survival.

The treatment effect is summarized in **Table 2**. PR was the best response, and an objective response was observed in two patients in the FP group and two patients in the RP group. The SD of the FP group was significantly higher than that of the RP group (82.1% vs. 47.8%, *P* = 0.01). The ORR of the whole population was 7.8% (4/51), the ORR of the FP group was 7.1% (2/28), and the ORR of the RP group was 8.7% (2/23). The DCR of the FP group (89.3%) was higher than that of the RP group (56.5%), and the DCR of the whole population was 74.5%. The median PFS of the FP group was 6.4 months (HR = 0.445; 95% CI: 5.527–7.273), and that of RP group was 3.9 months (HR = 0.594; 95% CI: 2.736–5.064). The difference was statistically significant (*P* = 0.0209, **Figure 2**).

**Table 2:** Curative effect evaluation.

Best overall response	Total, *n* (%)	FP (*n* = 28), *n* (%)	RP (*n* = 23), *n* (%)	*P-*value
Complete response	0	0	0	1
Partial response	4 (7.8)	2 (7.1)	2 (8.7)	0.709
Stable disease	34 (66.7)	23 (82.1)	11 (47.8)	**0.01**
Progressive disease	13 (25.5)	3 (10.7)	10 (43.5)	0.08
Objective response rate	4 (7.8)	2 (7.1)	2 (8.7)	0.709
Disease control rate	38 (74.5)	25 (89.3)	13 (56.5)	0.08

FP, fruquintinib combined with PD-1 inhibitors; RP, regorafenib combined with PD-1 inhibitors.

The bold values represent P < 0.05, and the difference is statistically significant.

**Figure 3:**
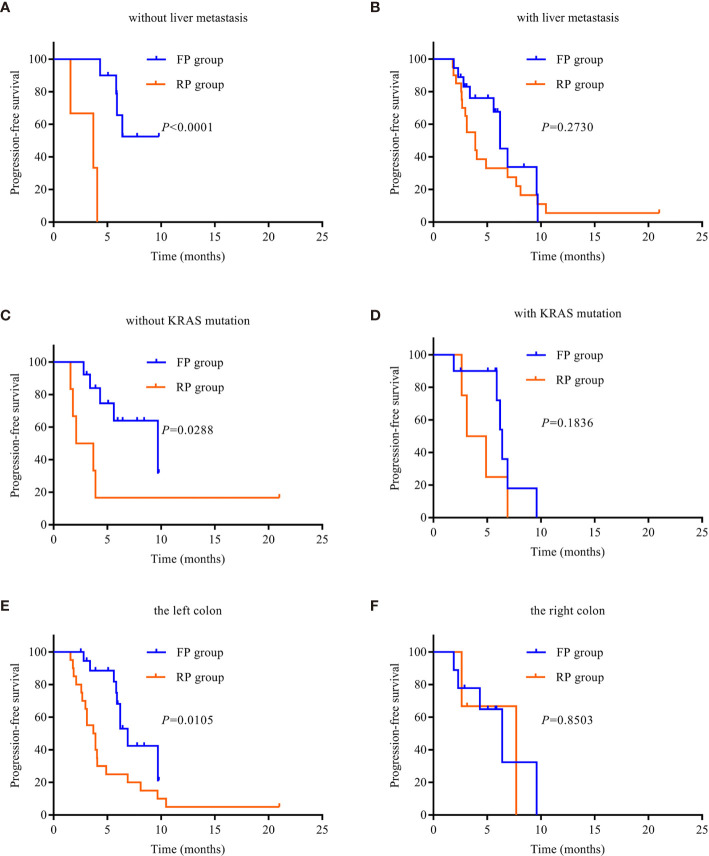
Kaplan–Meier survival curves. **(A)** PFS in patients without liver metastasis. **(B)** PFS in patients with liver metastasis. **(C)** PFS in patients without KRAS mutation. **(D)** PFS in patients with KRAS mutation. **(E)** PFS of the left colon. **(F)** PFS of the right colon. PD-1, programmed death receptor-1; FP, fruquintinib combined with PD-1 inhibitors; RP, regorafenib combined with PD-1 inhibitors; PFS, progression-free survival.

In order to analyze the beneficiaries of FP therapy compared with those of RP therapy, we performed Kaplan–Meier survival analysis and log-rank tests. In terms of patients with liver metastasis, there was a significant difference in median PFS between the FP and RP groups without liver metastasis (*P* < 0.0001, **Figure 3A**), but there was no significant difference in patients with liver metastasis (*P* > 0.05, **Figure 3B**). For patients with the wild type of KRAS, there was a significant difference in median PFS between the FP and RP groups (*P* = 0.0288, **Figure 3C**). For patients with the KRAS mutant, there was no significant difference between the two groups (*P* = 0.1836, **Figure 3D**). Based on the primary location of the tumor, there was a significant difference in median PFS between the FP and RP groups with the left colon as the primary location (*P* = 0.0105, **Figure 3E**) but not in the groups with the right colon as the primary location (*P* = 0.8538, **Figure 3**). In addition, there was no significant difference in median PFS between the FP and RP groups with or without peritoneal metastasis (*P* > 0.05, **Figures S1A, B**).

### Safety

Adverse events were evaluated in 28 patients in the FP group and 23 patients in the RP group. All patients experienced adverse events. The common treatment-related adverse events (AE) of any level in the FP group were liver dysfunction (42.8%), palmar–plantar erythrodysesthesia (39.3%), hypertension (35.7%), capillary endothelial hyperplasia (RCCEP) (39.3%), and proteinuria (32.1%). The common treatment-related AE of any level in the RP group were liver dysfunction (52.2%), palmar–plantar erythrodysesthesia (43.5%), hypertension (39.1%), RCCEP (39.1%), proteinuria (30.4%), and fatigue (30.4%). The incidence of liver dysfunction, palmar–plantar erythrodysesthesia, and hypertension in the RP group was higher than that in the FP group, but the difference was not statistically significant (**Table 3**). Grade 3 adverse events in the FP group were diarrhea (*n* = 1) and liver dysfunction (*n* = 2); there were no deaths due to adverse events. The adverse events related to grade 3 treatment in the RP group were palmar–plantar erythrodysesthesia (*n* = 1), rash (*n* = 1), liver dysfunction (*n* = 1), colonic perforation (*n* = 1), and myocardial enzyme elevation (*n* = 1); one patient died of immune myocarditis.

**Table 3:** Adverse events.

	All			Grade >3		
	FP group (*n* = 28)	RP group (*n* = 23)	*P*-value	FP group (*n* = 28)	RP group (*n* = 23)	*P-*value
*n* (%)	28 (100)	23 (100)	1	3 (10.7)	5 (21.7)	0.281
Palmar–plantar erythrodysesthesia	11 (39.3)	10 (43.5)	0.95	0	1 (4.3)	0.265
Hypertension	10 (35.7)	9 (39.1)	0.193	0	0	1
Fatigue	7 (25.0)	7 (30.4)	0.665	0	0	1
Rash	5 (17.8)	4 (17.30)	0.404	0	1 (4.3)	0.265
Capillary endothelial hyperplasia (RCCEP)	11 (39.3)	9 (39.1)	0.762	0	0	1
Proteinuria	9 (32.1)	7 (30.4)	0.358	0	0	1
Fever	2 (3.6)	1 (4.3)	0.673	0	0	1
Oral mucositis	2 (7.1)	1 (4.3)	0.238	0	0	1
Diarrhea	2 (7.1)	0	0.425	1 (3.6)	0	0.36
Decreased appetite	6 (21.4)	3 (13.0)	0.434	0	0	1
Liver dysfunction	12 (42.8)	12 (52.2)	0.575	2 (7.1)	1 (4.3)	0.673
Hyperthyroidism	1 (3.6)	0	0.36	0	0	1
Hypothyroidism	8 (28.5)	6 (26.1)	0.984	0	0	1
Platelet count decreased	5 (17.8)	2 (8.6)	0.493	0	0	1
Neutrophil count decreased	2 (7.1)	1 (4.3)	0.424	0	0	1
Hoarseness	1 (3.6)	0	0.36	0	0	1
Colonic perforation	1 (3.6)	2 (8.6)	0.529	0	1 (4.3)	0.265
Lipase elevated	0	0	1	0	0	1
Interstitial pneumonitis	0	0	1	0	0	1
Myocardial enzyme elevation	0	1 (4.3)	0.265	0	1 (4.3)	0.265

FP, fruquintinib combined with PD-1 inhibitors; RP, regorafenib combined with PD-1 inhibitors; RCCEP, capillary endothelial hyperplasia.

### Prognostic Factor Analysis

The prognostic factors affecting survival are shown in **Table 4**. Multivariate analyses showed that the comparison between fruquintinib and regorafenib was identified as an independent risk factor for two kinds of PFS (HR = 2.688; 95% CI: 1.246–5.797; *P* = 0.012).

**Table 4:** Univariate and multivariate analyses of risk factors for progression-free survival.

	Univariate analysis			Multivariate analysis		
	HR	95% CI	*P*-value	HR	95% CI	*P*-value
Age (years), (</≥65)	1.082	0.467–2.511	0.854			
Sex (female/male)	1.502	0.770–2.931	0.233			
Baseline ECOG PS (0/1/2)	**1.953**	**1.21–3.151**	**0.006**	2.17	1.259–3.74	0.05
First diagnosis time (months), (</≥18)	1.161	0.557–2.421	0.69			
Tumor location (left/right)	1.005	0.435–2.32	0.991			
Primary disease site at first diagnosis (colon/rectum)	0.763	0.393–1.478	0.422			
Liver metastasis (yes/no)	0.613	0.266–1.413	0.251			
Treatment lines (3/>3)	1.269	0.652–2.472	0.483			
Gene mutation status (RAS wild type/RAS mutant)	0.95	0.628–1.437	0.809			
Targeted drugs (fruquintinib/regorafenib)	**2.069**	**1.050–4.079**	**0.036**	**2.688**	**1.246–5.797**	**0.012**
Prior chemotherapy with VEGF (yes/no)	**2.999**	**1.052–8.545**	**0.04**	2.135	0.664–6.863	0.203
Prior chemotherapy with EGFR (yes/no)	**0.683**	**0.476–0.982**	**0.039**	0.962	0.632–1.464	0.856
PD-1 inhibitors	1.567	0.986–2.49	0.057			

ECOG PS, Eastern Cooperative Oncology Group performance status; HR, hazard ratio; CI, confidence interval; VEGF, vascular endothelial growth factor; EGFR, epidermal growth factor receptor.

The bold values represent P < 0.05, and the difference is statistically significant.

## Discussion

Immunotherapy is a promising treatment method for patients with mCRC. Based on several large trials, ICIs, including anti-PD-1 and CTLA-4 antibodies, have been approved by the US Food and Drug Administration for the treatment of MSI-H or dMMR mCRC patients (7, 9). However, MSS and pMMR CRC have a low immune response, and most MSS CRC patients do not benefit from ICIs alone (8, 34, 35).

Many studies are exploring anti-VEGF therapy combined with ICIs to overcome the drug resistance of pMMR CRCs. First, VEGF-driven angiogenesis can lead to the expansion of tumor-suppressing immune cells (including Tregs and MDSCs) and increase the infiltration of tumor-associated macrophages (TAMs) in the tumor site (36–38). Secondly, VEGF also exerts immunosuppressive effects by inhibiting progenitor cells differentiated with CD4+ and CD8+ lymphocytes (39); T-cell proliferation is decreased, and cytotoxicity is weakened. In addition, VEGF has been shown to increase T-cell failure by increasing the expression of PD-1, CTLA-4, TIM3, and LAG3 on T cells. This provides a strong theoretical basis for the combination of angiogenesis inhibitors and ICIs.

Fruquintinib and regorafenib are both antiangiogenic drugs and third-line treatments for advanced CRC (19, 21), but there is a lack of head-to-head clinical research on their efficacy. Indirect comparison of fruquintinib and regorafenib by meta-analysis showed that there was no significant difference in efficacy and safety between them (23–28). Preclinical studies have shown that fruquintinib combined with PD-1 inhibitors and regorafenib combined with PD-1 inhibitors have synergistic effects in a CRC model (32, 40). At present, there is no research report comparing the efficacy of FP and RP. Our retrospective study shows that FP has better survival benefits than RP in late mCRC.

In the REGONIVO study, 24 Japanese MSS mCRC patients were treated with regorafenib + nivolumab, the ORR was 36%, and the median PFS time was 7.9 months (29). The preliminary results provided MSS patients with refractory mCRC and oncologists with hope. However, a retrospective study of 18 MSS CRC patients at the American Cancer Center in 2019 failed to reveal the comparable clinical activity of regorafenib plus nivolumab, with a median PFS of 2.0 months (30). In a retrospective study, 23 Asian patients with MSS or pMMR mCRC treated with regorafenib combined with anti-PD-1 antibody had an ORR of 0% and a median PFS of 3.1 months (31). In our study, 23 MSS mCRC patients treated with regorafenib combined with PD-1 inhibitors had an ORR of 8.7% and a median PFS of 3.9 months, similar to those in the Asian population. The PFS (3.9 months) in our study was not as long as the PFS (7.9 months) reported in the REGONIVO study, but there was one patient in the RP group who had been taking medication for as long as 21 months. The data at the cutoff time showed SD, and the patient was still using RP. We need to further analyze the clinical data of the patient. However, the PFS (3.9 months) of the RP group was much better than the PFS (2.0 months) of the group in the US study, probably because the US study included only five Asian patients (27.8%). Different ethnic characteristics may also lead to differences in the efficacy of the combination.

The median PFS of the FP group was superior to that of the RP group in the treatment of MSS mCRC. The reasons may be as follows: first, fruquintinib belongs to a new generation of small molecular tyrosine kinase inhibitors with strong effects, which is highly selective to VEGFR-1, VEGFR-2, and VEGFR-3 but has no obvious inhibitory effect on other kinase activities; it is expected to maintain target inhibition and minimize toxicity (17, 18). Regorafenib is a multitarget kinase inhibitor (MKI), which inhibits the activation of VEGFR-1, VEGFR-2, VEGFR-3, FGFR, PDGFR, KIT, RET, TIE2, and BRAF (20). Second, the adverse reactions of inhibitors were more tolerable in the FP group than in the RP group. Although there was no significant difference in adverse reactions between the FP and RP groups, the adverse reactions in the FP group were generally lower than those in the RP group. Third, the proportion of liver metastasis in the FP group (64.3%) was lower than that in the RP group (87%). As an immunologically tolerant organ, the liver may reduce the intrahepatic and extrahepatic immune responses of tumor patients (41, 42). Fourth, the RP group was treated with regorafenib before the combined treatment, but the FP group was not treated with fruquintinib before the combined treatment. Notably, one patient in the FP group progressed 6.9 months after the use of regorafenib combined with sintilimab, and the regimen of fruquintinib combined with sintilimab still achieved good results. As of the data cutoff date, fruquintinib was used in combination with sintilimab for 5.06 months. Hence, in possible future trials, patients who have made progress when previously treated with regorafenib combined with immunosuppressants should not necessarily be excluded from receiving the combination of fruquintinib and immunosuppressant therapy.

Owing to its evolving immune tolerance, the liver is thought to be associated with a high proportion of immunosuppressive cells (41). Both primary liver cancer and liver metastasis can use liver immune tolerance to suppress the anticancer responses and weaken the efficacy of ICIs (42). In this study, we observed that the curative effect in the FP group was better than that in the RP group in patients without liver metastasis; the difference was statistically significant, but there was no significant difference in the liver metastasis groups. This result suggests that the FP regimen is more effective in advanced CRC patients without liver metastasis than in those with metastasis. The KRAS oncogene is one of the most common mutation genes in cancer. KRAS mutation has been found in approximately half of mCRC patients. KRAS mutation results in highly invasive tumor biology and poor prognosis (43–45). In the right colon, KRAS mutations are common (46). In this study, we observed that there was no significant difference in KRAS mutant and right tumor between the FP and RP groups; however, the curative effect in the FP group was better than that in the RP group for mCRC patients harboring the KRAS wild type and having the left colon as the primary location. These findings suggest that the FP regimen is relatively effective in advanced CRC patients with KRAS wild type and left tumor, but this needs to be confirmed in large-sample randomized studies.

This study has some limitations. First, this study is a single-center retrospective study, which inevitably has selection bias. Second, four different PD-1 inhibitors were used in this study, which affected the uniformity of the treatment process. Third, the number of cases was relatively small. Fourth, because of the late market time of fruquintinib, patients in the FP group began its use later than those in the RP group, and there may be a time bias. Fifth, the doses of fruquintinib and regorafenib were not uniform in patients, which further increased the heterogeneity of this study. Sixth, not all patients were tested for RAS and BRAF genes, which limited the analysis of their effects on the efficacy of drug therapy. Finally, the PD-L1 CPS and TMB of this study are unknown and cannot be used to determine the best population for immunosuppressant use. Therefore, the results of this study should be further extended to large-scale prospective studies in order to obtain a higher level of medical evidence.

In summary, FP has better survival benefits than RP. Patients with no liver metastasis, KRAS wild type, and left colon tumor may be the beneficiaries of FP. FP may become a new treatment option for advanced mCRC with MSS or pMMR.

## Data Availability Statement

The original contributions presented in the study are included in the article/**Supplementary Material**. Further inquiries can be directed to the corresponding author.

## Ethics Statement

Written informed consent was obtained from the individual(s) for the publication of any potentially identifiable images or data included in this article.

## Author Contributions

JW and YM designed the study. LS and YW collected the data. LS analyzed and interpreted the data. LS, SH, DL, YM, and JW carried out the clinical treatment and management of the patients. LS and JW prepared the final draft. All authors contributed to the article and approved the submitted version.

## Funding

This work was supported by the National Natural Science Foundation of China (No. 82060435).

## Conflict of Interest

The authors declare that the research was conducted in the absence of any commercial or financial relationships that could be construed as a potential conflict of interest.

## Publisher’s Note

All claims expressed in this article are solely those of the authors and do not necessarily represent those of their affiliated organizations, or those of the publisher, the editors and the reviewers. Any product that may be evaluated in this article, or claim that may be made by its manufacturer, is not guaranteed or endorsed by the publisher.

## Supplementary Material

Supplementary Figure 1 Kaplan-Meier survival curves. **(A)** PFS of patients without peritoneal metastasis. **(B)** PFS of patients with peritoneal metastasis.

## Abbreviations

PD-1, programmed death receptor-1; mCRC, metastatic colorectal cancer; MSS, microsatellite stability; pMMR, effective mismatch repair; FP, fruquintinib combined with PD-1 inhibitors; RP, regorafenib combined with PD-1 inhibitors; ICI, immune checkpoint inhibitor; ECOG PS, Eastern Cooperative Oncology Group performance status; mAb, monoclonal antibodies; dMMR, DNA mismatch repair defects; MSI-H, high microsatellite instability; CR, complete remission; PR, partial remission; SD, stable disease; PD, progression of disease; ORR, objective response rate; DCR, disease control rate; PFS, progression-free survival; OS, overall survival; CTC5.0, toxicity standard version 5.0; SE, standard deviation; HR, hazard ratio; CI, confidence interval; IQR, interquartile range; AE, adverse events; RCCEP, capillary endothelial hyperplasia; TAMs, tumor-associated macrophages; VEGF, vascular endothelial growth factor; EGFR, epidermal growth factor receptor.
