# Supplementary figures and images for: Efficacy and Safety of Fruquintinib Plus PD-1 Inhibitors Versus Regorafenib Plus PD-1 Inhibitors in Refractory Microsatellite Stable Metastatic Colorectal Cancer
Source: Front Oncol. 2021 Oct 6;11:754881. doi: 10.3389/fonc.2021.754881 (PMC8526894; doi:10.3389/fonc.2021.754881)

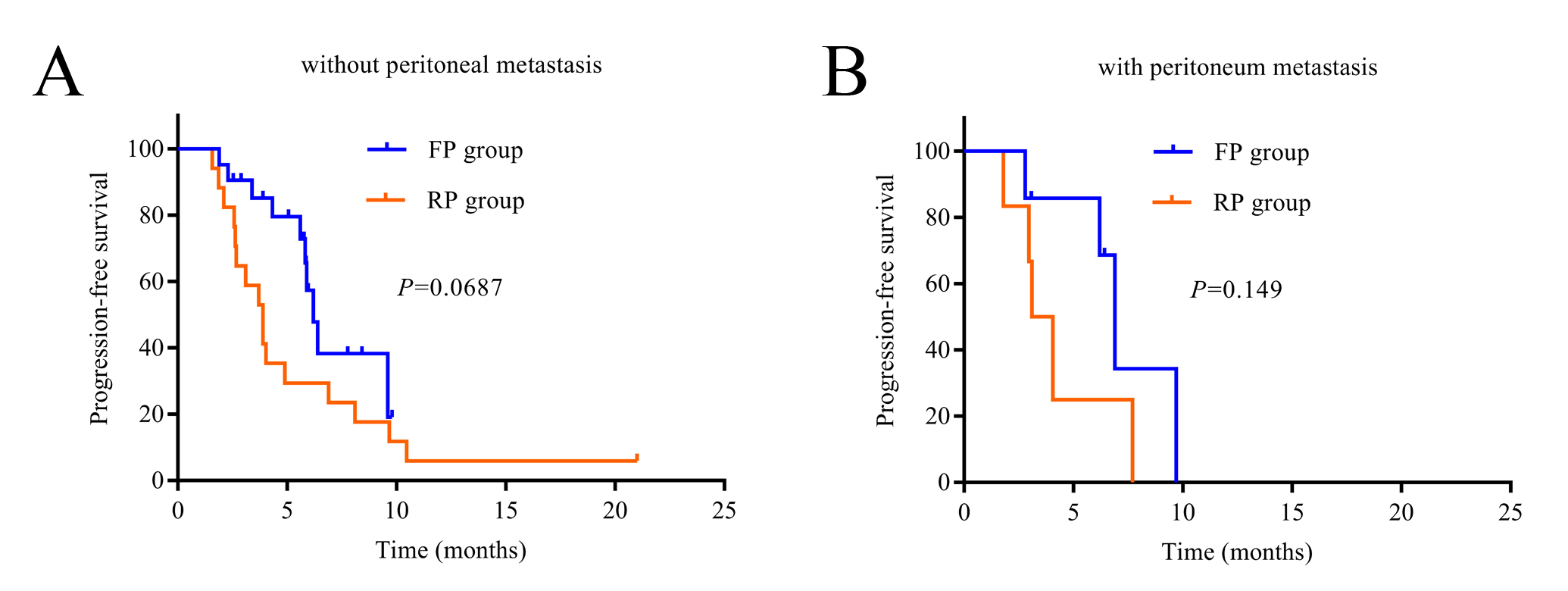

Supplement: Supplementary Figure 1 — Kaplan-Meier survival curves. (A) PFS of patients without peritoneal metastasis. (B) PFS of patients with peritoneal metastasis. [file Image_1.jpeg]
